# Supplementary material for: Kinematic characteristics of the tennis serve from the ad and deuce court service positions in elite junior players
Source: PLoS One. 2021 Jul 22;16(7):e0252650. doi: 10.1371/journal.pone.0252650 (PMC8297898; doi:10.1371/journal.pone.0252650)
Supplement: S1 Appendix — (PDF) [file pone.0252650.s002.pdf]

Appendix 1. Variables of interest.

Variables	Operational definition
Starting position	
Front foot position to baseline	Angle of front foot axis relative to baseline
Back foot position to baseline	Angle of back foot axis relative to baseline
Lateral feet distance	Distance between feet (measured from first metatarsal)
Upper torso position to baseline	Angle of upper torso relative to baseline
Preparation	
Front knee flexion	
Back knee flexion	
Trunk extension	
Trunk tilt	Lateral trunk flexion
Max. upper torso position	Maximum angle of upper torso relative to baseline
Counter-upper torso ROM	ROM between upper torso starting position and max upper torso position
Shoulder external rotation	
Elbow flexion	
Propulsion	
Front knee extension	Maximum angular velocity of front knee extension
Back knee extension	Maximum angular velocity of back knee extension
Trunk flexion	Maximum angular velocity of trunk flexion
Trunk tilt	Maximum angular velocity of trunk tilt
Shoulder internal rotation	Maximum angular velocity of shoulder internal rotation
Elbow extension	Maximum angular velocity of elbow extension
Wrist flexion	Maximum angular velocity of wrist flexion
Impact	
Front knee flexion	
Back knee flexion	
Trunk extension	
Trunk tilt	Lateral trunk flexion
Upper torso position to baseline	Angle of upper torso relative to baseline at impact
Upper torso ROM	ROM between max. upper torso position and upper torso position at impact
Shoulder abduction	
Elbow flexion	
Wrist extension	
Ball kinematics	
Ball velocity	Peak ball velocity of each stroke
Ball impact location X (lateral)	Relative to first metatarsal of the front foot at the instant of starting position (Positive x pointed lateral to the left (back-view))
Ball impact location Y (forward)	Relative to first metatarsal of the front foot at the instant of starting position (Positive y pointed away from the net)
Ball impact location Z (upward)	Relative to first metatarsal of the front foot at the instant of starting position (Positive z pointed vertically upwards)

Max: maximum, ROM: range of motion
