# Supplementary material for: Kinematic characteristics of the tennis serve from the ad and deuce court service positions in elite junior players
Source: PLoS One. 2021 Jul 22;16(7):e0252650. doi: 10.1371/journal.pone.0252650 (PMC8297898; doi:10.1371/journal.pone.0252650)
Supplement: S2 Appendix — (PDF) [file pone.0252650.s003.pdf]

S2 Appendix. Intra-session reliability statistics for each performance measure.

Variables		ICC (90% Confidence limit)		SEM (90% Confidence limit)	
**Starting position**					
Front foot position to baseline	[deg]	0.96	(0.92 - 0.98)	3.9	(3.3 - 4.8)
Back foot position to baseline	[deg]	0.79	(0.64 - 0.90)	4.4	(3.7 - 5.5)
Lateral feet distance §	[cm]	0.97	(0.95 - 0.99)	2.3	(2.0 - 2.9)
Upper torso position to baseline	[deg]	0.81	(0.67 - 0.91)	7.7	(6.5 - 9.5)
**Preparation**					
Front knee flexion	[deg]	0.94	(0.88 - 0.97)	4.3	(3.6 - 5.2)
Back knee flexion	[deg]	0.87	(0.77 - 0.94)	4.1	(3.5 - 5.1)
Trunk extension	[deg]	0.99	(0.98 - 0.99)	1.3	(1.1 - 1.6)
Trunk tilt	[deg]	0.96	(0.92 - 0.98)	1.4	(1.2 - 1.7)
Max. upper torso position	[deg]	0.92	(0.85 - 0.96)	3.2	(2.7 - 4.0)
Counter-upper torso rotation	[deg]	0.81	(0.67 - 0.91)	7.9	(6.7 - 9.9)
Shoulder external rotation	[deg]	0.93	(0.87 - 0.97)	3.2	(2.8 - 4.0)
Elbow flexion	[deg]	0.88	(0.79 - 0.95)	3.9	(3.3 - 4.8)
**Propulsion**					
Front knee extension ω	[deg/s]	0.71	(0.52 - 0.86)	66.8	(56.7 - 82.6)
Back knee extension ω	[deg/s]	0.76	(0.60 - 0.89)	52.7	(44.7 - 65.2)
Trunk flexion ω	[deg/s]	0.83	(0.70 - 0.92)	32.5	(27.5 - 40.1)
Trunk tilt ω	[deg/s]	0.89	(0.80 - 0.95)	36.7	(31.2 - 45.4)
Shoulder internal rotation ω	[deg/s]	0.73	(0.56 - 0.87)	185.3	(157.2 - 229.0)
Elbow extension ω	[deg/s]	0.86	(0.75 - 0.94)	133.2	(113.0 - 164.7)
Wrist flexion ω	[deg/s]	0.91	(0.83 - 0.96)	110.2	(93.5 - 136.3)
**Impact**					
Front knee flexion	[deg]	0.71	(0.52 - 0.86)	6.8	(5.8 - 8.4)
Back knee flexion	[deg]	0.58	(0.36 - 0.78)	6.6	(5.6 - 8.3)
Trunk extension	[deg]	0.93	(0.87 - 0.97)	2.8	(2.4 - 3.4)
Trunk tilt	[deg]	0.86	(0.75 - 0.94)	1.8	(1.6 - 2.3)
Upper torso position to baseline	[deg]	0.94	(0.89 - 0.98)	4.5	(3.8 - 5.5)
Upper torso rotation (ROM)	[deg]	0.96	(0.92 - 0.98)	4.8	(4.1 - 6.0)
Shoulder abduction	[deg]	0.88	(0.78 - 0.95)	2.6	(2.2 - 3.2)
Elbow flexion	[deg]	0.93	(0.87 - 0.97)	2.4	(2.0 - 3.0)
Wrist extension	[deg]	0.90	(0.82- 0.96)	2.4	(2.0 - 2.9)
**Ball kinematics**					
Ball velocity	[km/h]	0.95	(0.91 - 0.98)	4.7	(4.0 - 5.9)
Ball impact location X (lateral)	[cm]	0.89	(0.78 - 0.96)	9.5	(7.9 - 12.2)
Ball impact location Y (forward)	[cm]	0.76	(0.59 - 0.89)	10.9	(9.3 - 13.7)
Ball impact location Z (upward)	[cm]	0.97	(0.95 - 0.99)	4.3	(3.7 - 5.4)

§ first metatarsal of front foot to first metatarsal of rear foot; # range of motion between upper torso starting position and maximum upper torso position during preparation; ω maximum angular velocity
Reliability data calculated using spreadsheet for analysis of validity and reliability of Hopkins (Hopkins, Sportscience 19, 36-42, 2015)
