# Supplementary figures and images for: Kinematic characteristics of the tennis serve from the ad and deuce court service positions in elite junior players
Source: PLoS One. 2021 Jul 22;16(7):e0252650. doi: 10.1371/journal.pone.0252650 (PMC8297898; doi:10.1371/journal.pone.0252650)

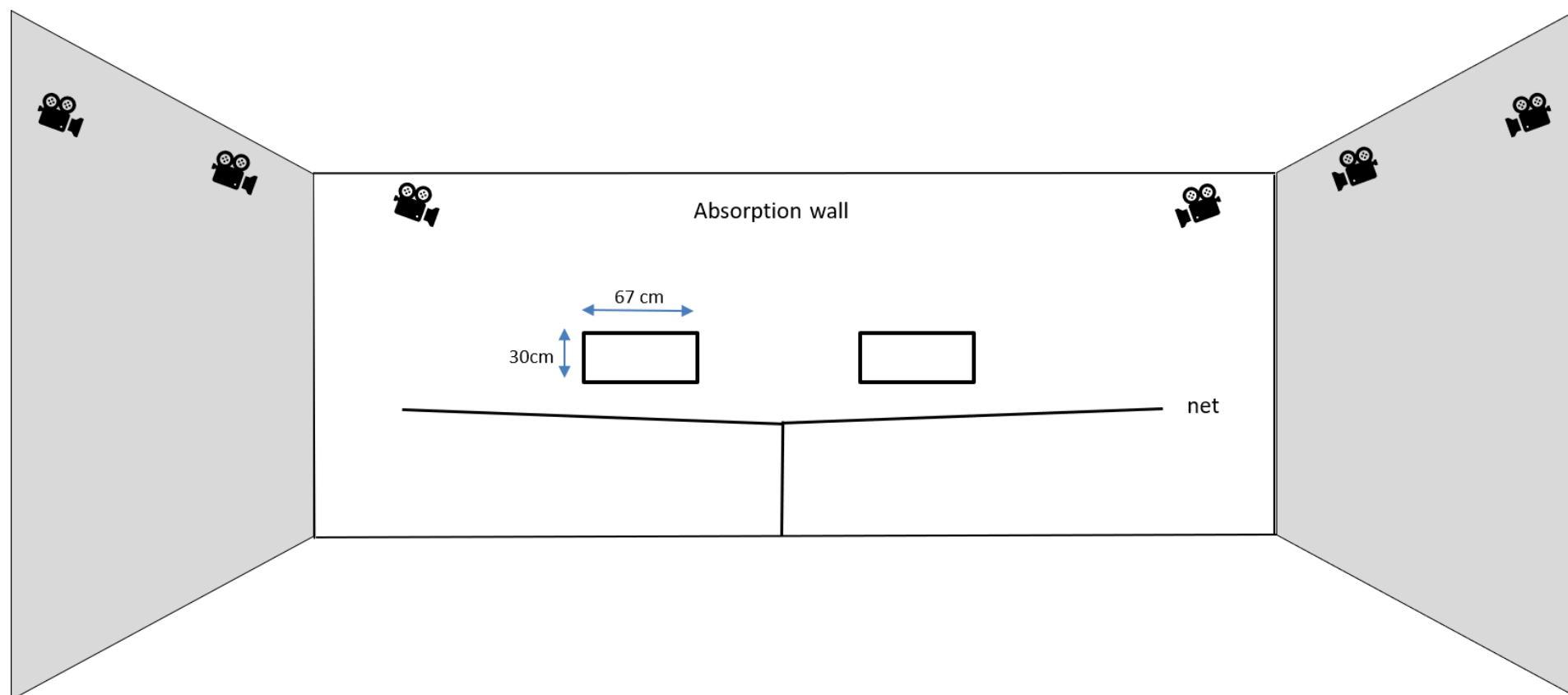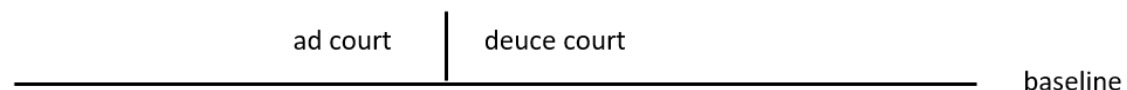

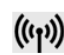 Radar gun (height: 3 m)

Supplement: S1 Fig — (PDF) [file pone.0252650.s004.pdf]
